# Report on the 1st Indian Cancer Congress 2013

**DOI:** 10.3332/ecancer.2013.ed32

**Published:** 2013-12-03

**Authors:** Richard Sullivan, Arnie Purushotham

**Affiliations:** 1 Kings Health Partners Cancer Centre, London, UK; 2 Institute of Cancer Policy, London, UK; 3 Kings College London, UK

The 1st Indian Cancer Congress (Delhi, 21-24th Nov) has been a reminder that the world of cancer is not all about the ASCOs and the ECCOs. What made this conference arguably more impactful and important globally than most others, was its relevance not just for India but also for many other emerging and low-income economies. Most cancer patients will live and die far away from high-income systems. Indeed India is pioneering in many domains of cancer research that high-income countries could and should learn from, from research into cost-effective site-specific care pathways to technological innovations.

In a country as socio-politically diverse as India it was a triumph to bring the four major associations (Association of Radiation Oncologists of India, Indian Society of Medical & Pediatric Oncology, Indian Association of Surgical Oncology and the Indian Society of Oncology) together for the first time. With over four thousand delegates the program reflected the richness and diversity of India. Plenary sessions, special tracts, and education jostled with round-tables on a huge range of topics. Coffee tables, seemingly ever open, hummed with old friends catching up, jocular arguments and new research relationships. It was dynamic, slightly chaotic but hugely intellectually stimulating.

The round tables on subjects such as “beyond tobacco control” and cost effective cancer care were hotbeds of debate. These policy debate sessions were excellent and far better than anything heard at other conferences. The conference had a strong focus on site-specific research with a really strong showing from the surgical community, another pleasant change from the dominant medical oncology nature of conferences in Europe and North America. Nursing and palliative care also had a strong showing which was excellent given that in India they both struggle for recognition.

Highlights? There were many and therefore it would be unfair to pick any one individual or discipline out. However, it is worth mentioning the National Cancer Grid (NCG) of India, now expanded to 36 cancer centres which had its third meeting at the Congress. There is little doubt that this is a huge step forward for tackling cancer in India. The NCG is undertaking, amongst other areas, issues of quality assurance for cancer care and the development of research infrastructure.

In a country where health is a mostly a state responsibility and where geographic variations are marked, the ability to bring together cancer leaders from across state boundaries has been a major achievement. The commitment by the NCG leaders to improving cancer care quality through evidenced-based guidelines, for example like those developed through Tata Memorial Centre reflects a reality, even on show at the congress, of a lack of governance around the quality of care in many places.

It was a privilege to attend this Congress and the organisers and participants should be congratulated for hosting a scientific meeting that has national and global impact.

Watch videos from the 1st Indian Cancer Congress for free

